# Post Hepatitis B vaccination sero-conversion among health care workers in the Cape Coast Metropolis of Ghana

**DOI:** 10.1371/journal.pone.0219148

**Published:** 2019-06-28

**Authors:** Dorcas Obiri-Yeboah, Yaw Asante Awuku, George Adjei, Obed Cudjoe, Anna Hayfron Benjamin, Evans Obboh, Daniel Amoako-Sakyi

**Affiliations:** 1 Department of Microbiology and Immunology, School of Medical Sciences, University of Cape Coast, Cape Coast, Ghana; 2 Department of Internal Medicine and Therapeutics, School of Medical Sciences, University of Cape Coast, Cape Coast, Ghana; 3 Department of Community Medicine, School of Medical Sciences, University of Cape Coast, Cape Coast, Ghana; 4 Department of Maternal and Child Health, School of Nursing and Midwifery, University of Cape Coast, Cape Coast, Ghana; University of Cincinnati College of Medicine, UNITED STATES

## Abstract

**Background:**

HBV vaccine is known to offer protection against transmission of HBV infection. Health care workers are mandated to have this vaccination as part of their occupational health safety measures. Post vaccination response data for HCWs in our setting is not available. This study therefore aimed to evaluate the anti-HBs titre levels after Hepatitis B vaccination among HCWs from selected heath facilities in the Cape Coast Metropolis, Ghana.

**Methods:**

A multicenter (3 selected sites) analytical cross-sectional study involving 711 HCWs was conducted. Five (5mls) of blood samples were collected from each study participant and the serum used for HBV immunological profile testing anti-HBs quantification by ELISA test (Fortress Diagnostics Limited, Northern Ireland, United Kingdom). Data analyses were performed using Stata version 14.0 software (STATA Corp, Texas USA).

**Results:**

The median age of participants was 29 years (IQR = 26–35 years). Majority (80.9%, n = 575) took their vaccination from Government health facilities compared with 19.1% (n = 136) from private vaccination sources. A total of 7 (3 males and 4 females) were found to be HBsAg positive giving prevalence of 1%. In all, 8.2% (n = 58) of the HCWs had anti-HBs titre levels <10IU/ml giving a sero-protection rate of 91.8%. HCWs who received 3 doses of HBV vaccine were more likely to be sero-protected as compared to those who received only one dose in multivariate analysis (aOR = 3.39, 95%CI: 1.08–10.67), p<0.037). Gender, cigarette smoking and alcohol consumption were not found to be associated with sero-protection.

**Conclusion:**

There is a high HBV vaccine efficacy among HCWs in the Cape Coast Metropolis of Ghana with higher prevalence of anti-HBs titre level associated with full vaccine dose adherence. Post vaccination antibody titre determination could be an integral part of HBV vaccination protocol for HCWs in Ghana.

## Introduction

Hepatitis B virus (HBV) infection is highly prevalent around the globe and causes significant morbidity and mortality. The latest World Health Organization (WHO) global estimates suggests that 258 million are living with HBV infections. The estimates further suggests that almost 900,000 people died from HBV related complication such as cirrhosis and hepatocellular carcinoma in 2015 [1]. The public health implication of HBV is further complicated by a marked geographic heterogeneity in the burden of disease. For instance, whereas the WHO Western Pacific and African Regions have the highest adult population prevalence of 6.2% and 6.1%, respectively, the WHO European Region has only 0.7% of its adult population infected with HBV [1]. These estimates are regional; national and district perspectives often shows further geographic heterogeneity and this is exemplified by several studies including a meta-analysis study in Ghana that reported an HBsAg seropositivity of 12.3% [2].

Besides variations in the geographical distribution of HBV, the risk of infection is known to vary among occupations. Health care workers (HCWs) represent one of the largest high risk groups for HBV infection worldwide and are at four times greater risk compared to the general adult population [3]. Thus, it was unsurprising to find that interventions made by the Centres for Disease Control and Prevention (CDC) in 1997 to vaccinate all healthcare workers has significantly decreased the seroprevalence of HBV infection in this target group globally [4–6]. The vaccine is generally administered intramuscularly in the deltoid region at three doses of 0-month, 1-month, and 6-month schedules. Post vaccination serologic testing for antibody to hepatitis B surface antigen (Anti-HBs) is recommended 1–2 months after the last vaccine dose for HCWs who are at risk for occupational exposures [7]. However, in some HCWs (non-responders), there is vaccine failure. Approximately 5-10% of those vaccinated against HBV fail to respond with the development of antibody and moreover, anti-HBs titres decrease over time. Reasons for non-response to HBV vaccination might be multifactorial including host factors such as age, smoking, obesity, gender, and host genetics; and vaccine and vaccination factors such as vaccine type, vaccination dose, injection site, and the time passed after the last vaccination [8,9]. Anti-HBs titre is used to evaluate the efficacy of hepatitis B vaccine and titres of >10 mIU/ml is considered protective [10]. Studies have demonstrated that HBV vaccine induced protection persists for at least 11 years and even up to 30 years [11–15]. Healthcare workers who have been vaccinated against HBV infection and do not develop immunity remain at a high risk of being infected [11] and chances of spread of infection may be increased if infection control measures are not strictly followed.

Despite HBV infection being a major healthcare issue in both community and healthcare settings in Ghana, there is no comprehensive data regarding sero-conversion after HBV vaccination among healthcare workers in the country; hence there is little evidence of the efficacy of the vaccine, the sero-conversion rate and duration of immunity among vaccinated HCWs in Ghana. This ominous lack of post vaccination surveillance programs in Ghana makes the detection and management of vaccine non-responders difficult. In the specific case of HBV vaccination in Ghana, multiple vaccines sources, byzantine supply chains, and weak regulatory frameworks makes the assessment of vaccine effectiveness an uphill task. However, it is imperative to establish surveillance programmes to detect antibody decline and vaccine failure, particularly, among HCWs who are at a higher risk of infection. In view of their heightened exposure and risk to infection, the HCW population constitute a logical starting point for efforts to decipher HBV vaccine failure and non-respondance. This study therefore aimed to evaluate the sero-conversion rate after HBV vaccination among HCWs from selected heath facilities in the Cape Coast Metropolis, Ghana.

## Materials and methods

### Ethical considerations

The Cape Coast Teaching Hospital Ethical Review Board approved this study. In addition, the study procedures were explained to each participant after which written informed consent was obtained by signing to participate in the study. In addition, potential participants were assured that they can refuse to participate in the study without attracting any punishment. Confidentiality of study participants was ensured by identifying them with unique study codes. A specific arrangement was made to ensure that HCWs who are found to be infected with HBV from this study are linked to specialist care at the viral hepatitis and Gastroenterology clinics at CCTH. In addition, those found to need boosters received counseling from qualified professionals on the options available to them.

### Study design and participants

This was an analytical cross-sectional study conducted at the Cape Coast Teaching Hospital (a tertiary facility), Cape Coast Metropolitan Hospital (a regional hospital), and University Hospital (district level facility). Together, these facilities serve the residents of the Cape Coast Metropolis in the Central Region, Ghana. Recruitment and sample collection was done between July and November 2018 and all staff of participating hospitals who had patient contact, a history of HBV vaccination, and consented were eligible for inclusion into the study. Eligible HCWs who self-reported HBV positivity were excluded from the study. Appointments were made with the various units of the participating hospitals and HCWs present were given the opportunity to participate in the study. This process was iterated a number of times at different work schedules until the sample size was obtained. Socio-economic and demographic data were obtained with a standardized and pre-tested questionnaire developed by the researchers for this study. The questionnaire gathered information from HCWs on their hepatitis B vaccination history (e.g. vaccine doses received, schedule, booster vaccination) and clinical data (e.g. pre vaccination HBsAg testing and the results).

### Sample collection and laboratory analysis

Five (5mls) of blood were collected from each study participant into EDTA vacutainer tubes and transported to the School of Medical Sciences (SMS) laboratory at the University of Cape Coast (UCC) for processing and testing. Samples were separated by centrifugation and the serum used for HBV immunological profile testing following the manufacturers protocol (Guangzhou Wondfo Biotech Co., Ltd., China) and the rest was kept at -20°C and later used to estimate HBV antibody titre quantification.

The presence of anti-HBs was determined using the anti-HBs quantitative ELISA kit (Fortress Diagnostics Limited, Northern Ireland, United Kingdom). Each test was performed in duplicates according to the manufacturer’s instructions, the plate was read on ELISA plate reader Emax (Molecular Devices LLC, USA).

### Sample size considerations

Data on the prevalence of HBV among healthcare workers is not readily available in our setting and thus, the prevalence of HBV vaccination among health workers was estimated to be 50%. Hence with 95% confidence level, significance level of 0.05, 3% margin of error and a total healthcare worker population of 1500, a minimum of 625 health workers are required to be enrolled in this study. Making a provision of 10% for contingencies, a sample size of 688 health workers is required for this study. Stata version 14.0 was used for the sample size calculation.

The data were analyzed with Stata version 14.0 (STATA Corp, Texas USA). The distribution of data was ascertained by determining how normal or skewed the data was and this eventually determined whether parametric or non-parametric statistics was employed. Appropriate measures of central tendencies and dispersion were used for descriptive analysis. Seroprevalence of HBsAg among respondents was estimated. Geometric mean anti-HBs titres together with its 95% confidence interval among respondents was calculated. Proportion was also used to estimate the prevalence of anti-HBs among respondents. Then chi-squared test, independent t-test, bivariate and multivariate logistic regressions where done to determine the association of factors with the main outcome of this study (sero-conversion status). Independent variables that are established by literature to influence the outcome were used as a priori for multivariate logistic regressions in addition to all variables with p-values ≤0.20 in the bivariate regression. All statistical tests in this analysis were two-tailed and p-values less than 0.05 were considered to be statistically significant.

## Results

### Sociodemographic characteristics of participants

**Fig 1:**
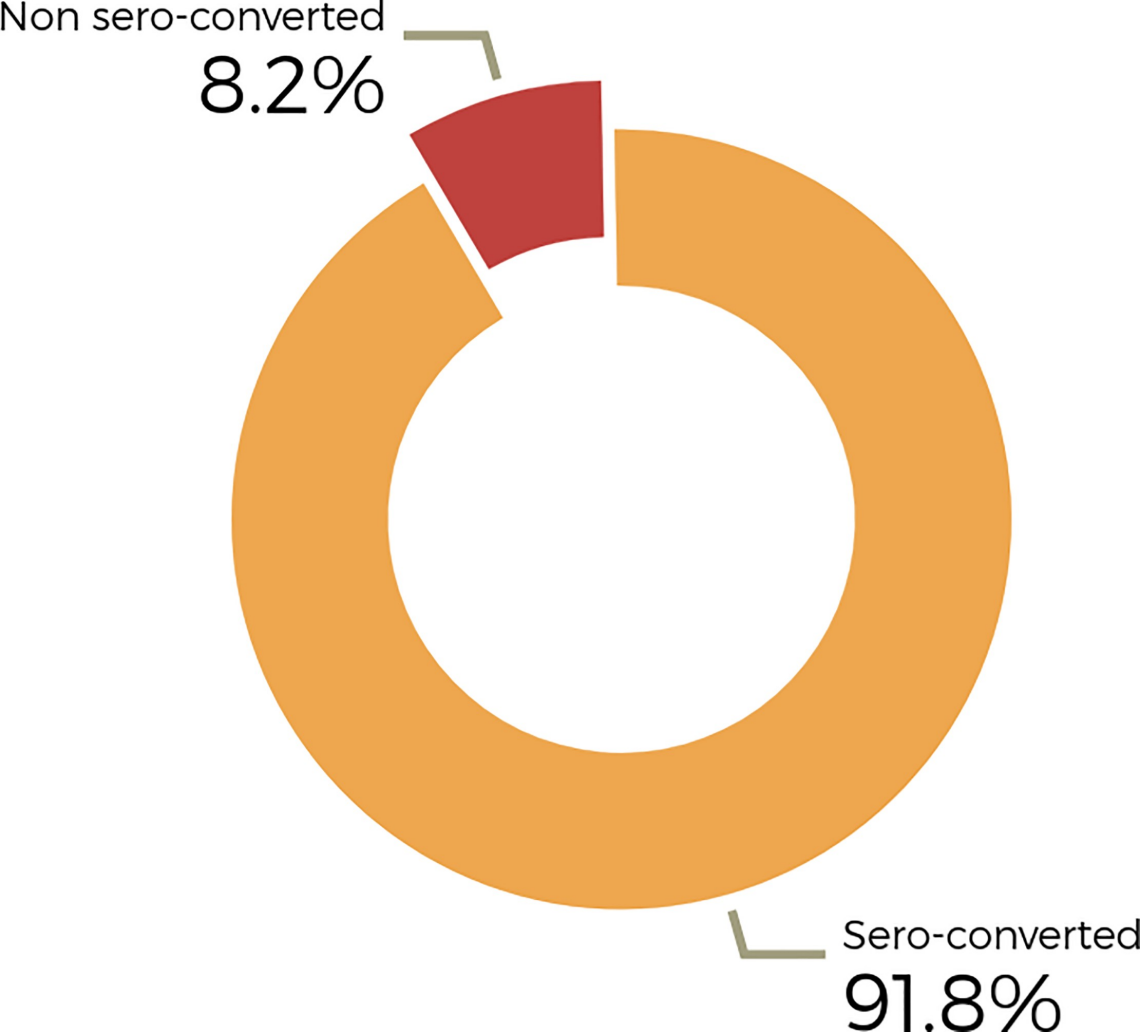
Sero-protection (Anti-HBs titre level >10IU/ml) rate among 711 HCWs.

The median age of participants was 29 years (IQR: 26–35 years). Majority of participants were in the Nurse/Midwife/Health Assistants category (62.3%, n = 443) and 64% (n = 455) have worked for <5 years. Most participants (80.9%, n = 575) took their HBV vaccinations from government facilities and only 7.2% (n = 51) had ever taken a booster since completing their vaccinations. A total of 7 HCWs (3 males and 4 females) were found to be HBsAg positive yielding a prevalence of 1% (Table 1). Among participants, a total of 58 (8.2%) HCWs had anti-HBs titre levels <10IU/ml (Fig 1).

**Table 1:** Sociodemographic and other relevant characteristics of study participants (N = 711).

Variable	Median/Frequency (n)	Percentage (%)/Interquartile Range Range
Age^*^, years (N = 695)		
Median	29.0	26.0–35.0
20–30	412	59.3
31–40	213	30.6
>40	70	10.1
**Sex**		
Male	251	35.3
Female	460	64.7
**Current Department**		
Internal Medicine and Therapeutics	175	24.6
Obstetrics and Gynaecology	126	17.7
Child Health	48	6.7
Surgery	89	12.5
Laboratory	53	7.5
Public Health Unit	26	3.7
Other (e.g. Records, sanitation etc.)	194	27.3
**Occupation**		
Doctors/Physician assistants	83	11.7
Nurse/Midwife/Health Assistants	443	62.3
Lab Technicians/Technologists/etc.	49	6.9
Disease Control Staff	7	1.0
Pharmacy Staff	6	0.8
Other (e.g. Records, sanitation and other support staff)	123	17.3
**Years of practice**		
Median	3.0	1.5–6.0
<5	455	64.0
5–10	245	34.5
>10	11	1.5
**Source of HBV vaccination**		
Government facility	575	80.9
Private facility	136	19.1
**HBV vaccine booster ever taken**		
Yes	51	7.2
No	660	92.8
**How long since vaccination, (yrs)**		
<5	348	48.9
5–10	359	50.5
>10	4	0.6
**HBV surface antigen seroprevalence prevalence**		
Positive	7	1.0
Negative	704	99.0

*Data on age was available for only 695 participants

### Factors associated with sero-conversion among participants

Occupation (p = 0.042), HBV vaccine dose received by HCWs (p<0.001) and HCWs who complied with dose interval (p<0.001) were found to be associated with sero-protection status of HCWs. However, age (p = 0.820), sex (p = 0.312), years of practice (p = 0.382), source of HBV vaccination (p = 0.300), years after last vaccination had been received by HCWs (p = 0.452) and HBV vaccine booster received by HCWs (p = 0.112) were not associated with sero-protection status (Table 2).

**Table 2:** Association between socio-demographic and other characteristics of HCWs and sero-protection status.

Variable	Sero-protection		
	Yes, n (%)	No, n (%)	p-value
**Age (years)**			0.820
20–30	381 (92.5)	31 (7.5)	
31–40	194 (91.1)	19 (8.9)	
>40	64 (91.4)	6 (8.6)	
**Gender**			0.312
Male	227 (90.4)	24 (9.6)	
Female	426 (92.6)	34 (7.4)	
**Occupation**			***0*.*042***
Doctors/Physician assistants	79 (95.2)	4 (4.8)	
Nurse/midwife/Health assistants	408 (92.1)	35 (7.9)	
Lab technicians/technologists	40 (81.6)	9 (18.4)	
Other	126 (92.7)	10 (7.3)	
**Years of practice**			0.382
<5	421 (92.5)	34 (7.5)	
5–10	221 (90.2)	24 (9.8)	
>10	11 (100.0)	0 (0.0)	
**Source of HBV vaccination**			0.300
Government facility	531 (92.3)	44 (7.7)	
Private facility	122 (89.7)	14 (10.3)	
**How long ago was the last vaccination (years)?**			0.452
<5	319 (91.7)	29 (8.3)	
5–10	331 (92.2)	28 (7.8)	
>10	3 (75.0)	1 (25.0)	
**HBV vaccine dose received**			***<0*.*001***
1	25 (73.5)	9 (26.5)	
2	70 (85.4)	12 (14.6)	
3	558 (93.8)	37 (6.2)	
**Vaccination interval**			0.680
0,1,2 months	374 (91.7)	34 (8.3)	
0,1,6 months	255 (92.4)	21 (7.6)	
Not sure	24 (88.9)	3 (11.1)	
**Complied with dose interval?**			**<0.001**
Yes	583 (94.0)	37 (6.0)	
No	55 (78.6)	15 (21.4)	
Not sure	15 (71.4)	6 (28.6)	
**Taken a booster since schedule completion**			0.112
Yes	50 (98.0)	1 (2.0)	
No	603 (91.4)	57 (8.6)	
**Smoke cigarette**			0.401
Yes	5 (83.3)	1 (16.7)	
No	648 (91.9)	57 (8.1)	
**Drink alcohol**			0.819
Yes	116 (91.3)	11 (8.7)	
No	537 (92.0)	47 (8.0)	

Male HCWs had the lower Geometric Mean Titre (GMT) value of 96 (95%CI: 76.6–120.4) compared with 131.6 (95%CI: 113.4–152.9) for females. In addition, those who took their vaccination from private sources and those who had never taken a booster also had lower GMT values. There was progressively higher GMT values (45.7, 68.8 and 113.8) for those who took only 1, 2 or 3 doses of the HBV vaccine, respectively. Participants who smoke cigarettes and those who drink alcohol were also found to have lower GMT values in their categories (84.1 vs. 118.1 and 96.8 vs. 122.9), though this was not statistically significant (Table 3).

**Table 3:** Geometric Mean Titre (GMT) value of healthcare workers (N = 711).

Variable	GMT	95% CI	p-value
**Age**			0.532*
20–30	125.3	106.3–147.6	
31–40	106.6	84.0–135.3	
>40	120.3	83.3–173.7	
**Sex**			0.028**
Male	96.0	76.6–120.4	
Female	131.6	113.4–152.9	
**Occupation**			0.667*
Doctors/PAs	122.6	83.5–179.9	
Nurse/Midwife/Health Assistants	119.1	101.6–139.5	
Lab Technicians/Technologists etc.	88.1	48.4–160.6	
Other	123.1	94.5–160.4	
**Source of HBV vaccination**			0.813**
Government facility	118.6	103.3–136.2	
Private facility	114.2	84.4–154.4	
**How long ago was the last vaccination (years)**			0.058*
<5	123.2	102.4–148.1	
5–10	114.0	96.0–135.5	
>10	41.2	2.0–843.5	
**HBV vaccine dose received**			<0.001*
1	45.7	20.2–103.4	0.695^α^
2	68.8	42.1–112.6	0.002^β^
3	113.8	118.2–151.5	0.001^γ^
**Vaccination interval**			0.845*
0,1,2 months	114.1	96.2–135.3	
0,1,6 months	122.8	101.0–149.2	
Not sure	124.6	65.9–235.5	
**Complied to dose interval**			<0.001*
Yes	133.7	118.3–151.1	<0.001^α^
No	51.3	29.2–90.2	1.00 ^β^
Not sure	44.0	14.8–130.1	0.009 ^γ^
**Taken a booster**			0.003**
Yes	232.0	177.0–304.1	
No	111.7	97.8–127.6	
**Smoke**			0.627**
Yes	84.1	10.8–654.2	
No	118.1	104.1–134.0	
**Drink alcohol**			0.153**
Yes	96.8	69.4–135.0	
No	122.9	107.4–140.6	

^α^ = p-value (HBV vaccine dose received: 1 versus 2; Complied to dose interval: Yes versus No)

^β^ = p-values (HBV vaccine dose received: 2 versus 3; Complied to dose interval: No versus Not sure)

^γ^ = p-values (HBV vaccine dose received: 1 versus 3; Complied to dose interval: Yes versus Not sure)

* = overall p-value for oneway ANOVA test

** = p-value for t-test

**Fig 2:**
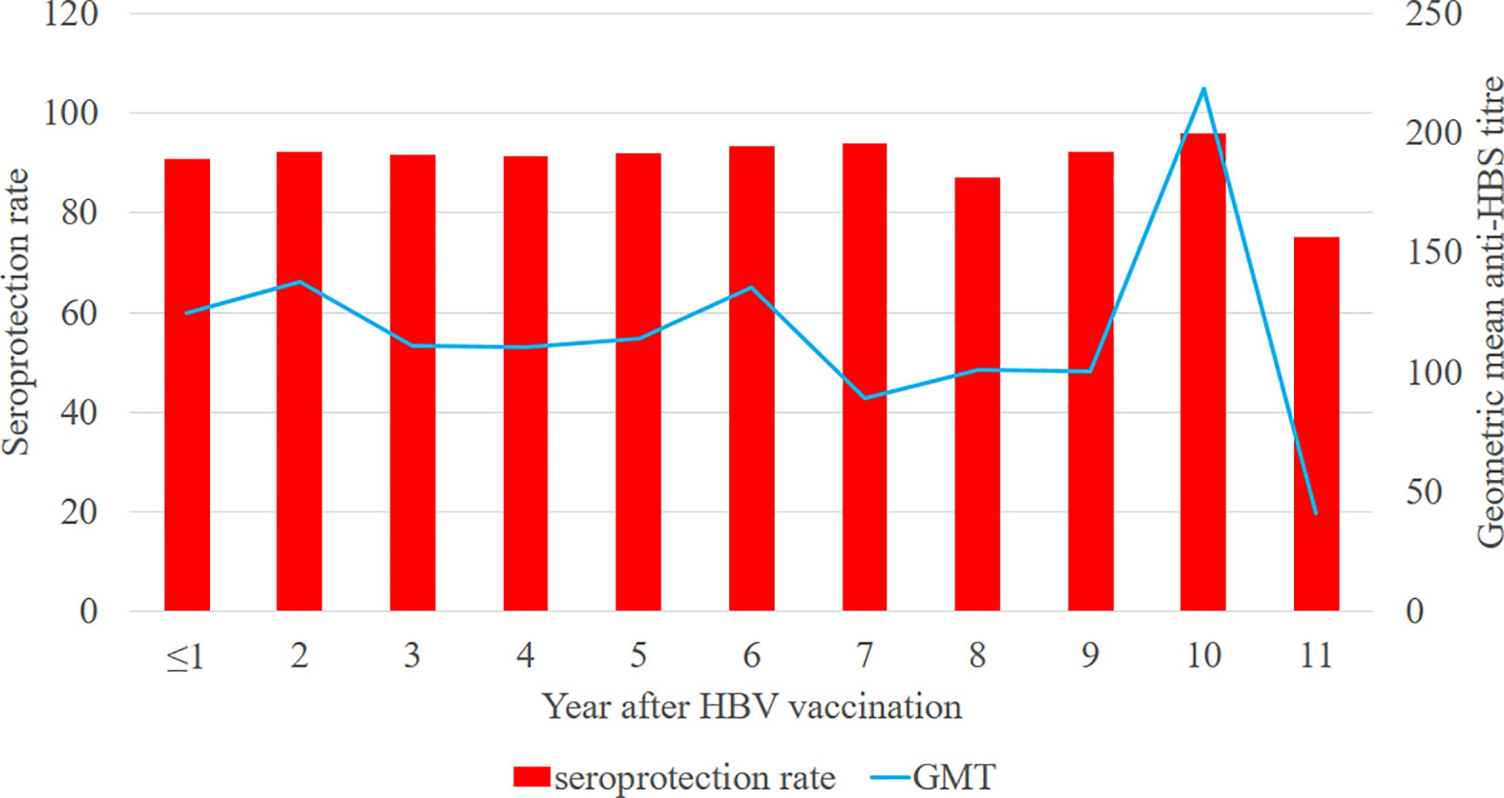
Distribution of sero-protection rate and Anti-HBs titre 1–11 years post vaccination.

The sero-protection rate had a generally upward trend from 1 year (90.9%) post vaccination to 7 years (93.8%) with a fall to 87.2% by 8 years. The highest rate and GMT value (96.0% and 218.4) was observed at 10 years post vaccination and the lowest at 11 years (75.0% and 41.2), Fig 2.

HBV vaccine doses received by HCWs had influence on sero-protection status (likelihood ratio p<0.001). HCWs who received 3 doses of HBV vaccine were 3.39 times more likely to be sero-protected as compared to those who received only one dose of the vaccine (aOR = 3.39, 95%CI: 1.08–10.67), p<0.037). Neither a HCW’s occupation nor having a booster shot were statistically significant in the multivariate model (Table 4)

**Table 4:** Predictors for sero-protection status of healthcare workers.

	Bivariate Regression		Multivariate Regression	
Variable	OR (95% CI)	p-value	aOR (95% CI)	p-value
Occupation				
Doctors/PAs	1		1	
Nurse/Midwife/Health Assistants	0.59 (0.20–1.71)	0.331	0.64 (0.22–1.89)	0.420
Lab Technicians/Technologists etc.	0.23 (0.07–0.78)	0.018	0.23 (0.06–0.83)	0.024
Other	0.64 (0.19–2.10)	0.460	0.86 (0.25–2.92)	0.805
**HBV vaccine dose received**				
1	1		1	
2	2.10 (0.79–5.58)	0.137*	1.86 (0.63–5.50)	0.264
3	5.43 (2.36–12.47)	<0.001*	3.39 (1.08–10.67)	0.037
**Complied to dose interval**				
Yes	1		1	
No	0.23 (0.12–0.45)	<0.001*	0.44 (0.17–1.13)	0.088*
Not sure	0.16 (0.06–0.43)	<0.001*	0.16 (0.05–0.44)	<0.001*
**Taken a booster**				
Yes	1		1	
No	0.21 (0.03–1.56)	0.128	0.27 (0.04–2.02)	0.201

*Likelihood ratio p<0.01

## Discussion

As a first step towards addressing the dearth of information on HBV post-vaccination immunity and vaccine efficacy in Ghana, this study evaluated the sero-conversion rate after HBV vaccination among HCWs from selected heath facilities in the Cape Coast Metropolis, Ghana. Over 91% of study participants showed evidence of post-vaccination immunity measured as anti-HBs titre level >10IU/ml. This level is higher than found among HCWs in the study by Mariki *et al* in Cameroun [16]. Although this sero-conversion rate is consistent with that reported by Sahana *et al* [17], further stratified analysis of sero-conversion within sub-population revealed interesting findings. For instance, relative to other staff categories (especially, doctors and PAs), sero-conversion rate was lower among Lab technicians/technologist. They also had the lowest anti-HBV geometric mean titres of 88.1 (48.4–160.6). Data from this study does not proffer reasons for this observation but the fact that this cadre of HCWs who routinely handle blood sample may not be adequately protected for HBV exposure is worrying. It has been demonstrated that some who do not respond to the HBV vaccination initially, when identified and re-vaccinated, produced antibodies with anti-HBs titre level >10IU/ml [18,19].

Demographic factors such as age and sex which are deemed to influence immune response did not influence sero-conversion and this was consistent with other studies [16]. Similar studies found age to be associated with sero-conversion rate among participants, with older ones having lower response rate [19–21]. This is most likely because such studies classified older adults as above 60 years [22] whereas a number of studies including this current one recruited participants of the younger adults population.

This study confirmed that receiving the 3 doses is essential for having and maintaining as high antibody titre levels. Vaccine dose and vaccination regimen are technical attributes that are fine-tuned through rigorous study designs and trials to optimize immune response. The need for booster dose after completion of the 3 doses has remained controversial with conflicting findings from various studies. While some concluded that taking a booster is necessary for maintaining adequate antibody titre levels [17], others found otherwise [13,18] and some studies were inconclusive on this issue [23]. In this study, though the geometric mean titre level was higher among those who had received a booster (p = 0.0003), multivariate analysis did not find an association between taking a booster and sero-protection rate (p = 0.201). Another study concluded that the antibody levels though decreased progressively over time, there was enough until after 10 years which supports the recommendation of taking booster every 10 years [19] especially among high risk groups like HCWs. In this study the antibody level rose to a high level with corresponding high sero protection rate until after the 10^th^ year post vaccination. The issue of booster doses and the interval at which it was taken, was not clear in this study thus making it difficult to examine the impact on antibody titre level.

The prevalence of HBsAg positivity (1%) found in this study among HCWs who had received at least 1 dose of vaccination, was high relative to reports from USA, comparable to findings from a study in Libya which found prevalence of 1.3% [24]. Although Ghana is in the WHO Africa region where HBV infection rate is estimated to be >10% by many studies [2,25], vaccination coverage particularly among HCWs is generally below expectation [26]. Such low coverage was found among HCWs in a study in Cameroun [16] and generally across Africa [27,28]. This situations requires urgent attention to reduce the vulnerability of these unprotected HCWs since vaccination has proven in many settings to reduce the prevalence of HBV infection [24,29]. The cost of vaccine as a challenge to access must be addressed to ensure increased coverage [30,31]. To ensure universal access to HBV vaccine in Ghana there must be a good collaboration with government, Global alliance for Vaccine Initiative (GAVI) and other supporting agencies. Further analysis of the data revealed that of the 7 participants who were found to be infected, 1 (12.3%) person had taken only 1 dose of the vaccine while 2 (28.6%) had taken 2 vaccine doses. Four (57.1%) had taken all 3 vaccine doses with 1 among them even taking a ‘booster’ dose. The fact that this study only had self-reporting of HBV screening before vaccination limits the interpretation of this finding. However, this finding would suggests that HCWs must be supported to take all 3 vaccines and also that, post vaccination titre measurement might be essential to enable none responders to be identified and appropriate interventions implemented.

This study has some limitations. These include the fact that other predictors of non-response like immunosuppression [32] were not investigated in this study. Self-reporting of HBsAg testing prior to vaccination and the vaccination history among HCWs has a limitation of recall bias. The need for proper vaccination records keeping is essential and would be a more objective evidence. While a longitudinal study would produce more definitive results, the funding was not available for that. Despite these, this study has brought to the fore important findings.

## Conclusion

There is a high HBV vaccine efficacy among HCWs in the Cape Coast Metropolis of Ghana with higher anti-HBs titre level associated with full vaccine dose adherence. Post vaccination antibody titer determination could be an integral part of HBV vaccination protocol for HCWs in Ghana.

## Supporting information

S1 Dataset This is the complete dataset for the manuscript. (XLSX)
